# Near-Complete Whole-Genome Sequencing of Two Burkholderia pseudomallei Strains Harbouring Novel Molecular Class D Beta-Lactamase Genes, Isolated from Malaysia

**DOI:** 10.1128/mra.00468-22

**Published:** 2022-08-31

**Authors:** Ummu Afeera Zainulabid, Norirwanisyam Mohamad Zain, Janaki Arumugam, Norhidayah Kamarudin, Muhammad ‘Adil Zainal Abidin, Abdullah Shamshir Abd Mokti, Noorlina Nordin, Ahmad Faizal Rakawi, Aman Shah Abdul Majid, Gnanasekaran Ashok, Alex Lourdes Francis, Darren Dean Tay, Natesan Vijayalakshami, How Soon Hin, Hajar Fauzan Ahmad

**Affiliations:** a Kulliyyah of Medicine, International Islamic University of Malaysia, Jalan Sultan Ahmad Shah, Kuantan, Pahang, Malaysia; b Hospital Raja Permaisuri Bainun, Jalan Raja Ashman Shah, Ipoh, Perak, Malaysia; c Hospital Tengku Ampuan Afzan, Jalan Tanah Putih, Kuantan, Pahang, Malaysia; d Faculty of Medicine, Quest International University, Ipoh, Perak, Malaysia; e Faculty of Industrial Sciences and Technology, Universiti Malaysia Pahang, Lebuhraya Tun Razak, Gambang, Pahang, Malaysia; University of Maryland School of Medicine

## Abstract

Here, we present two 7.1- and 7.3-bp near-complete genome sequences of Burkholderia pseudomallei strains of HTAA077 and HRPB058, isolated from a pus culture from a confirmed melioidosis patient at Hospital Tengku Ampuan Afzan, Kuantan, Malaysia, and from blood culture from a patient at Hospital Raja Permaisuri Bainun, Ipoh, Malaysia, using a Nanopore MinION instrument.

## ANNOUNCEMENT

Burkholderia pseudomallei is a pathogenic saprophyte that causes melioidosis (1). The disease is endemic in Malaysia, with various clinical presentations (2, 3), and its management includes 6 months of antibiotic regimen administration (4). Clinical specimens were collected from patients suspected of bacteremia in accordance with ethical approvals (International Islamic University Malaysia [IIUM] Research Ethics Committee [IREC] and National Medical Research Register [NMRR] via IREC 2021-193, NMRR-17-3238-39448, and NMRR-21-360-58751). The blood was cultured using a BD Bactec FX blood culture system (Becton, Dickinson and Company, NJ), and both samples were subcultured on blood agar media (Bio-Rad, CA) at 37°C to obtain a pure culture. The genomic DNA of the strains was extracted as described previously using the GenElute bacterial genomic DNA kit (Sigma-Aldrich Co., St. Louis, USA) (5). For the library preparation, DNA size selection using SPRIselect (Beckman Coulter Inc., IN) was performed to retain fragments larger than 6 kb. Approximately 1,000 ng of size-selected DNA as measured by a QFX fluorometer (Denovix Inc., Wilmington, USA) was used directly for library preparation using the ligation sequencing kit SQK-LSK110 (Oxford Nanopore Technologies [ONT], Oxford, UK). The prepared library was sequenced on a Flongle flow cell R9.4.1 (Oxford Nanopore Technologies) and base-called used the Guppy v5.0.7 “super accuracy model.” The raw fastq reads were length filtered to retain reads longer than 2 kb, followed by *de novo* assembly using the Flye v2.9 –nano-hq model (6). The assembled genome was subsequently polished with one round of RACON (https://github.com/isovic/racon) and then one round of Medaka (https://github.com/nanoporetech/medaka) (7), generating the final consensus assembly that consists of 2 circular sequences, which the genome automatically circularized with Flye, but not rotated and closed contigs. Genome completeness was subsequently assessed using BUSCO5 (https://gitlab.com/ezlab/busco) based on the Burkholderiales_odb10 lineage database (8). Genome annotation was carried out using the NCBI Prokaryotic Genome Annotation Pipeline (PGAP) with the GeneMarkS2-v.1.14_1.25 method (9), and the presence of acquired antibiotic resistance genes (ARGs), virulence factors, and human pathogenicity was analyzed using the program VirulenceFinder 2.0 (10) and ResFinder 2.1 (11). The HTAA077 strain has a total length of 7,276,308 bp, an average GC content of 68%, and ONT reads with a mean length of 67,923 and an *N*_50_ length of 4,082,678 bp. It contains 3 contigs, comprising 6,193 coding sequences with 76 RNAs. In addition, the HRPB058 strain has a total length of 7,094,056 bp, an average GC content of 68.1%, and ONT reads with a mean length of 32,1475 and an *N*_50_ length of 3,961,310 bp. It contains 2 contigs, comprising 5,897 coding sequences with 76 RNAs and 5,897 encoded proteins. Both virulence factors found in this isolate are related to antibiotic resistance genes of *bla*_OXA-43_, *bla*_OXA-57_, and *bla*_OXA-59_ that encoded an unknown beta-lactam group.

### Data availability.

The complete genome sequences of Burkholderia pseudomallei are available in the NCBI SRA database under accession numbers SRR18556255 (HTAA077) and SRR18556256 (HRPB056), and the BioProject number is PRJNA821438. The BioSample accession numbers for HTAA077 and HRPB058 are SAMN27097645 and SAMN27097644, respectively. The NCBI PGAP genome annotation numbers are GCA_024506485.1 (JANIED010000001) and GCA_024506415.1 (JANIED010000002) for HTAA077 and HRPB058, respectively.
